# Petroleum as source and carrier of metals in epigenetic sediment-hosted mineralization

**DOI:** 10.1038/s41598-019-44770-7

**Published:** 2019-06-04

**Authors:** Nicolas J. Saintilan, Jorge E. Spangenberg, Massimo Chiaradia, Cyril Chelle-Michou, Michael B. Stephens, Lluís Fontboté

**Affiliations:** 1 Department of Earth Sciences, University of Geneva, Rue des Maraîchers 13, 1205 Geneva, Switzerland; 2 Institute of Earth Surface Dynamics, University of Lausanne, Building Geopolis, 1015 Lausanne, Switzerland; 3 Formerly Geological Survey of Sweden (SGU), Box 670 SE-751 28, Uppsala, Sweden; 4 Division of Geosciences, Luleå University of Technology, SE-971 87 Luleå, Sweden; 5 Present Address: Institute of Geochemistry and Petrology, Department of Earth Sciences, ETH Zürich, Clausiusstrasse 25, 8092 Zürich, Switzerland

**Keywords:** Mineralogy, Geochemistry

## Abstract

Sediment-hosted ore deposits contribute a significant amount (up to 65%) of the global resources of lead and zinc. Among them, the Mississippi-Valley type deposits and related oil fields often comprise large-scale hydrothermal systems where regional host rocks are stained with disseminated liquid petroleum (crude oil) and other organic compounds. Current models for the formation of those epigenetic Pb-Zn sulphide deposits consider that metals are mostly leached from basement rocks and their detrital erosional products, and transported by oxidized basinal hydrothermal fluids as chloride complexes. Sulphide precipitation mainly occurs when these basinal brines interact with fluids rich in reduced sulphur species produced mostly by thermochemical sulphate reduction (TSR) mediated by hydrocarbons. Here, using organic geochemistry and Pb isotopes, we provide evidence that petroleum and associated water were key for the formation of sulphide mineralization in the world-class sandstone-hosted ore deposit at Laisvall, not only by supplying reduced sulphur but also by contributing metals in significant amounts. The lead originally found in bitumen of the Alum Shale Formation was transported —during an arc-continent collisional event— by liquid petroleum and associated water to the site of sulphide mineralization. The alteration of petroleum by TSR made lead available for precipitation as sulphide. The petroleum-associated lead represents 40 to 60% of the metal budget in the deposit, the remainder being sourced by leaching of basement rocks.

## Introduction

Metals contained in epigenetic sediment-hosted Zn-Pb sulphide deposits, which are referred as or related to Mississippi Valley-type (MVT) deposits, are thought to be mostly leached from local basement rocks and their detrital erosional rocks, and transported as chloride-metal complexes in oxidized basinal brines^1–4^. Base metal sulphide mineralization mainly takes place when the metal-bearing fluids mix with reduced fluids rich in sulphur species^3,4^. Organic compounds are commonly involved in the processes concurring to the accumulation of reduced sulphur species^3,4^. Several MVT ores are associated with rests of pre- to syn-ore altered organic matter (e.g., solid bitumen), which was involved in the production of reduced sulphur by thermochemical sulphate reduction (TSR)^4–10^.

The role of organic acids as transport agents of Pb and Zn in basinal fluids has been evaluated through analysis of metal concentrations in oil field brines and thermodynamic modelling^2,3^. Inverse correlation between concentration of organic acid anions (e.g., acetate) and metal content was noticed in basinal chloride-dominated and metal-bearing fluids^2^. Thus, in such brines, the minor complexation capability of organic acid anions (e.g., carboxylates) for Pb and Zn dismissed the possibility that organic acids play a role in the transport of Pb and Zn^3^. In the present study, we do not focus on the metal-bearing basinal brines but on the other mixing end-member(s) for which petroleum and the associated aqueous phases are involved^4–10^. Besides the established role of such organic compounds in the production of reduced sulphur for mineralization, their possible contribution to the metal pool for sulphide mineralization has only been conceptualized and tested experimentally^11,12^. Indeed, several metals (e.g., Ni, V, Mo, Zn, Cu, and Pb) are present in liquid petroleum at concentrations up to several hundreds of parts per million, similar to those related to metal-chloride complexes in basinal brines^12–14^. Therefore, potentially, petroleum compounds involved in precipitation of sulphide minerals may have also supplied metals.

## Connection between Sandstone-Hosted Pb-Zn Mineralization, Hydrocarbons and Alum Shale

**Figure 1:**
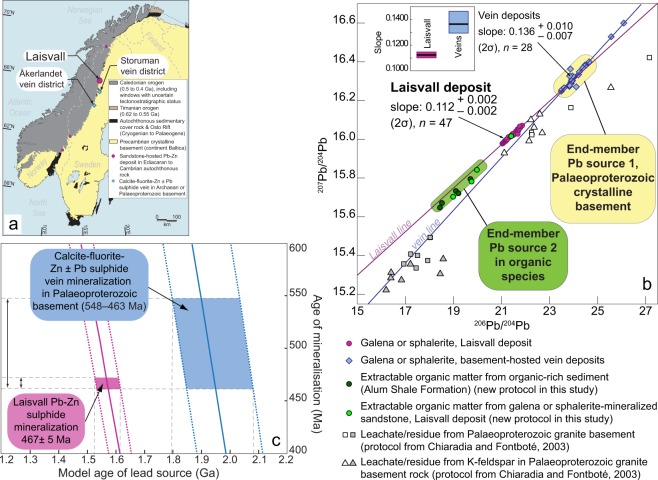
Lead sources in the epigenetic sandstone-hosted Pb-Zn deposit at Laisvall, Sweden. (**a**) Simplified geological map of Scandinavia showing the location of sandstone-hosted Pb-Zn sulphide deposits in Ediacaran to lower Cambrian autochthonous sandstone and calcite-galena vein occurrences in Palaeoproterozoic crystalline basement close to the current erosional front of the Caledonian orogen. (**b**) ^207^Pb/^204^Pb vs. ^206^Pb/^204^Pb plot showing the isotopic composition of (i) Pb-Zn sulphides from the Laisvall deposit (Supplementary Data Table SI1) and in basement-hosted calcite-galena vein occurrences (Storuman and Åkerlandet districts in Fig. 1a; Supplementary Data Table SI2), (ii) residue and leachate fractions from granite basement samples from the Laisvall area and their corresponding K-feldspar aliquots (Supplementary Data Table SI3) (ii) extractable organic matter (EOM) from shale hydrocarbon source rocks of the Alum Shale Formation and from the two mineralized sandstone horizons in the Laisvall deposit (Supplementary Data Table SI4), (**c**) Model age of lead source vs. timing of mineralization between 400 and 600 Ma for the Laisvall deposit and the calcite-galena vein occurrences in Palaeoproterozoic basement.

**Figure 2:**
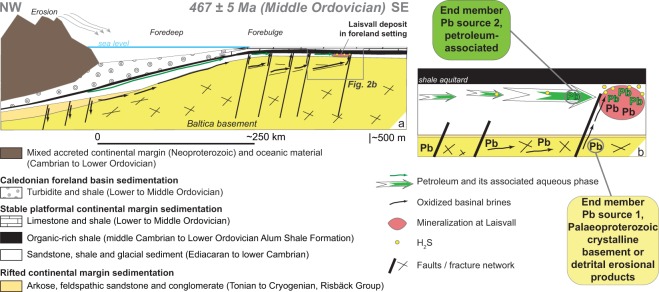
Geodynamic setting for mineralization at Laisvall bearing “petroleum-associated lead” and “basement-derived lead”. (**a**) Genetic model and palaeotectonic setting during mineralization in the Middle Ordovician at Laisvall. (**b**) Mineralizing processes and details of the two lead sources with emphasis on the low-radiogenic lead source supplied by petroleum and petroleum-associated brines. Petroleum also contributed H_2_S for mineralization via thermochemical sulphate reduction (modified after ref.^16^). Figure 2 is modified after a published figure with permission from “Springer Nature: Mineralium Deposita, vol. 51, A refined genetic model for the Laisvall and Vassbo Mississippi Valley-type sandstone-hosted deposits, Sweden: constraints from paragenetic studies, organic geochemistry, and S, C, N, and Sr isotope data, Saintilan, N.J., Spangenberg, J.E., Samankassou, E., Kouzmanov, K., Chiaradia, M., Stephens, M.B., and Fontboté, L., p. 639–664, Copyright, 2016”.

The world-class Laisvall MVT deposit (Fig. 1a; 64 Mt at 4% Pb, 0.6% Zn, 9 g/t Ag) in northern Sweden was mined for 40 years and was the largest Pb producer in Europe. Mineralization is found in two discrete ore horizons within a sedimentary sequence deposited on the stable, platformal margin to the continent Baltica during the Ediacaran to Early Ordovician^15,16^ (Fig. 2): (1) the Pb ± Zn Lower Sandstone, and (2) the Zn ± Pb Upper Sandstone. Sphalerite and galena appear in three forms: (1) interstitially between partly dissolved quartz grains (mainly in the Upper Sandstone Orebody), (2) replacing barite cement (mainly in the Lower Sandstone Orebody), or (3) replacing ore-stage pyrite in both orebodies^16^.

Epigenetic sulphide mineralization formed during the Middle Ordovician (467 ± 5 Ma) as a result of fluid migration triggered by early Caledonian orogenic activity related to an arc-continent collision^15^. Geological constraints and organic geochemistry of black shale and both mineralized and barren sandstone samples indicate that hydrocarbons migrated over several tens of kilometres from the middle Cambrian to Lower Ordovician Alum Shale Formation source rocks buried in the Caledonian foredeep into permeable Ediacaran to Cambrian sandstone in the forebulge setting^16,17^ (Fig. 2a). These hydrocarbons produced reduced sulphur species by TSR in sandstone traps^16–18^.

The sulphur isotope composition (δ^34^S values V-CDT) of galena (+21 to +27‰), sphalerite (+21 to +34‰) and ore-stage pyrite (+21 to +30‰) suggests that most H_2_S in the mineralizing fluid was produced by TSR from pore water seawater sulphate (+27 to +33‰) and barite cement (+14 to +33‰). Petrographic evidence from the layering of the Zn-Pb ore, sulphur isotope data, and occurrence of oxidized hydrocarbons (e.g., solidified as bitumen intergrown with sphalerite), collectively suggest that TSR was most probably confined to a hydrocarbon-water interface^16^. This interpretation is in line with the necessity of a physical contact between hydrocarbon and aqueous sulphate for TSR to occur given the fact that sulphate does not react by TSR in the solid state^19^. Recent experimental data in the 200° to 250 °C temperature range show that that the rates of *in situ* TSR reactions in ore deposits may be much faster than previously thought, in particular if thermally labile organo-sulphur compounds (e.g., thiols) are present and sulphide precipitation has taken place^20–22^.

The Alum Shale Formation contains hydrocarbon source rocks made of algal and bacterial kerogen^23^ with high contents of total organic carbon (up to 22 wt% TOC), Pb (up to 200 ppm) and other metals (e.g., Ni, V, Mo, U, Zn)^24^. The transport of base metals in petroleum depends on whether a metal preferentially partitions into the organic phase (e.g., V, Ni and Co in metalloporphyrins) or into the aqueous one (e.g., Pb and Zn chlorides)^13,25^. Lead, originally found in petroleum source rocks, might be present in the organic phase, but at much lower concentrations than, e.g., V, Ni and Co. However, Pb might be preferentially partitioned as inorganic complexes in the petroleum-associated waters (e.g., 2.1 mg/L Pb in petroleum vs. 111 mg/L Pb in the associated waters)^13,25,26^. Therefore, petroleum compounds produced by the Alum Shale rocks could have potentially supplied lead for sulphide mineralization in sandstone, adding to the established source of radiogenic lead derived from Palaeoproterozoic basement rocks at Laisvall or their detrital erosional products due to leaching by basinal brines^27–29^. In this study, we provide direct evidence in support of this hypothesis and show the significant contribution of “petroleum-associated lead” to the ore site.

## Lead Isotopic Composition of Pb-Zn Sulphides, Pb Model Ages and Sources of Lead

Calcite-galena veins, which occur in neighbouring districts to Laisvall and are hosted in the same Palaeoproterozoic Baltica basement province as that underlying the Laisvall district (Fig. 1a), illustrate the classical example of a metallogenic process involving leaching of metals solely from basement rocks^1,30^. In a conventional ^207^Pb/^204^Pb vs. ^206^Pb/^204^Pb diagram (Fig. 1b), the analysed Pb-Zn sulphides at Laisvall define a trend (“Laisvall line”) that is distinct from the isotopic composition of the Pb-Zn sulphides from the basement-hosted veins (“vein line”). Indeed, the slope of 0.112 ± 0.002 (2σ) for the “Laisvall line” is distinct within uncertainties from the slope (0.136 ± 0.010, 2σ) of the “vein line” (inset in Fig. 1b). These results suggest that the discrete Laisvall trend, which is different from the vein trend, is not compatible with leaching of metals solely from basement rocks and their erosional products.

We calculated lead source model ages (T) for a range of possible mineralization ages (t) between 400 and 600 Ma, consistent with the published Rb-Sr sphalerite isochron ages for the Laisvall deposit (467 ± 5 Ma)^15^ and the broadly coeval vein occurrences (548 to 463 Ma)^15,31^. The equation of the slope (*m*) of a palaeoisochron is:

1 $$m={(\frac{{}^{207}{\rm{Pb}}}{{}^{206}{\rm{Pb}}})}^{\ast }=\frac{1}{137.818}\times (\frac{{e}^{{{\rm{\lambda }}}_{235}{\rm{T}}}\,-{e}^{{{\rm{\lambda }}}_{235}{\rm{t}}}}{{e}^{{{\rm{\lambda }}}_{238}{\rm{T}}}-{e}^{{{\rm{\lambda }}}_{238}t}})$$

where λ_235_ and λ_238_ are the decay constants for ^235^U and ^238^U, respectively, and the asterisk symbolizes the radiogenic fraction.

Since galena (PbS) and sphalerite (ZnS) contain virtually no uranium and thorium, their lead isotopic composition approximates the common lead composition at the time of their precipitation. For vein mineralization ages between 400 and 600 Ma, the source of lead in the vein occurrences (*m* = 0.136) has a model age between 1.8 and 2.1 Ga (Fig. 1c), which is consistent with the geological age of the crystalline rocks in the basement province^32^. In contrast, considering the Laisvall mineralization age of 467 ± 5 Ma, the Laisvall trend would require a lead source with a significantly younger model age between ~1.52 and ~1.61 Ga (Fig. 1c), which is inconsistent with any known rock unit in the part of the Baltica basement province around the deposit. This result indicates that the Laisvall line is not a palaeoisochron but a mixing line between two discrete lead sources (“Laisvall mixing line”), a high-radiogenic lead end-member and a low-radiogenic lead end-member (Fig. 1b). The high-radiogenic lead end-member of the “Laisvall mixing line” is reasonably represented by a Pb isotopic composition similar to that of Pb-Zn sulphides in the basement-hosted vein-type mineralization (“end-member 1” in Fig. 1b). We suggest that this lead end-member corresponds to the isotopic composition of Pb leached from basement rocks at the time of vein formation (broadly coeval with the formation of the Laisvall deposit).

In order to test this hypothesis, we carried out experimental leaching of samples from granitic basement rocks at Laisvall (protocol from ref.^33^). We analysed separately leachate and residue fractions of whole rocks and K-feldspars. The results show that residues, which represent the common Pb isotope composition, fall on the low-radiogenic continuation of the “vein line” whereas leachates scatter at variably more radiogenic values around the “vein line” (Fig. 1b). This supports the contention that the Pb isotope spread of the “vein line” results from leaching of basement rocks at the time of the vein formation (i.e., 400–600 Ma ago) by basinal brines, as recognized in the widely accepted genetic model for MVT and related deposit types^4,16^. The fact that not all leachates fall on the regression line including vein Pb may be due to two reasons: (i) leachates are not corrected for time-integrated decay since the formation of the vein mineralization (this would variably shift isotopic compositions of leachates to the left along isochrons corresponding to 400–600 Ma ages, depending on their U/Pb ratios); Fig. 1b); (ii) the leaching procedure used is probably not fully representative (e.g., in terms of acidity, temperature, salinity of the experimental acid solution used) of the leaching of basement rocks by basinal brines 400 to 600 million years ago.

## “Petroleum-Associated Pb” in Epigenetic Pb-Zn Sulphide Mineralization

In order to test whether the low radiogenic end-member (“end-member 2” in Fig. 1b) could correspond to Pb initially associated with petroleum, a protocol was developed to obtain the lead isotopic composition of the organic solvent-extractable organic matter (bitumen) in Alum Shale rocks (*n* = 5) and mineralized sandstone samples (*n* = 4). In addition, four other mineralized sandstone samples and three subaliquots of the Alum Shale sample, which had the highest bitumen content, were utilized to determine the metal concentrations (i.e., U, Th, Pb, Zn) of the bitumen fractions (Supplementary Data Table SI5).

The Alum Shale samples have high bitumen contents (0.3 to 1.0 mg/g rock), compared to the mineralized sandstone samples (0.03 to 0.09 mg/g rock). The bitumen fractions in the shale samples were rich in asphaltenes and resins (NSO compounds). The hydrocarbon fraction mainly consisted of *n*-alkane, methyl-alkanes, and acyclic isoprenoids^16^. In the mineralized sandstone samples, the bitumen fractions contain neither U nor Th but significant Zn and Pb concentrations, in particular in the Lower Sandstone (LS) samples (Zn from 41 to 24,125 ppm, Pb between 3 and 400 ppm; Supplementary Data Table SI5). The bitumen fractions of the Alum Shale samples contain between 370 and 455 ppm U, ca. 200 ppm Zn, but are almost devoid of Th (2 ppm). We could thus safely conclude that the bitumen fraction in mineralized sandstone samples, which was derived from petroleum produced by the Alum Shale Formation^16–18^ (Fig. 2), had neither U nor Th that could contribute radiogenic Pb. The low Pb contents in the bitumen fraction of the Alum Shale samples would be compatible with the release of Pb from Alum Shale source rock to the petroleum and its associated aqueous phase before their migration. In light of this evidence, we can investigate if lead originally found in the bitumen of Alum Shale Formation was brought to the mineralization site at Laisvall as “petroleum-associated Pb”.

The five Alum Shale bitumen fractions have a lead isotopic composition that falls on the Laisvall mixing line on the low-radiogenic side (“end-member 2” in Fig. 1b). The lead isotopic composition of the bitumen fractions from three mineralized sandstone samples falls in the field of end-member 2 defined by the Alum Shale bitumen fractions. The bitumen fraction of the fourth mineralized sandstone sample has a Pb isotopic composition that is similar to the isotopic composition of Pb-Zn sulphides at Laisvall and therefore falls on the Laisvall mixing line. Our results imply that lead originally found in the bitumen of the Alum Shale rocks was transported eastwards during the arc-continent collisional event by liquid petroleum and its associated aqueous phase to the mineralization site in sandstone at Laisvall^15,16^ (Fig. 2b). This petroleum-associated lead was subsequently made available for precipitation as sulphides when the organic phase was altered by thermochemical sulphate reduction causing a change of the pH and Eh conditions.

## Implications for the Role of Petroleum as Ore Fluids and the Fate of Petroleum Reservoirs

Based on the above discussion, simple mixing equations (Table 1) using the average isotopic compositions of “end-member 1” (basement-derived Pb) and “end-member 2” (“petroleum-associated Pb”) suggest that low-radiogenic lead released by Alum Shale may have contributed between 40 and 60% of the total Pb budget of the Laisvall deposit. This “petroleum-associated Pb” accumulated originally in the organic-matter-rich and metal-rich Alum Shale during sedimentation and early diagenesis. This lead was subsequently incorporated into petroleum and associated aqueous phase during organic matter maturation. The remainder “basement-derived Pb” was provided by crystalline basement rocks or detrital erosional products leached by basinal brines^16,27,34^. At 467 Ma, in coincidence with orogenic activity, metal-rich petroleum and associated fluids migrated to a trap site at Laisvall, where they mixed with oxidized basinal brines containing basement-derived Pb. In presence of TSR-derived reduced sulphur species, lead and zinc sulphides precipitated to form the Laisvall deposit with Pb of bimodal origin.

**Table 1:** Estimation of the contribution in lead from the two end-members identified in the present study: (1) basement-derived lead and (2) “organic lead”.

Average isotopic composition	^206^Pb/^204^Pb	2σ	^207^Pb/^204^Pb	2σ		
End-member Pb source 1 (*n* = 25)	24.1513	0.0023	16.3337	0.0016		
End-member Pb source 2 (*n* = 5)	18.9976	0.0018	15.7135	0.0015		
“Extreme” ore samples on the “Laisvall line” (*n* = 47)	^**206**^ **Pb/** ^**204**^ **Pb**	**2σ**	^**207**^ **Pb/** ^**204**^ **Pb**	**2σ**	**%Pb contributed by end-member Pb source 2 (“organic lead”)**	**%Pb contributed by end-member Pb source 1 (“basement lead”)**
Aliquot in bottom left-hand corner	21.0369	0.0020	15.9777	0.0015	60%	40%
Aliquot in top right-hand corner	21.8592	0.0021	16.0710	0.0015	44%	56%

The estimation is based on the average lead isotopic composition (^206^Pb/^204^Pb and ^207^Pb/^204^Pb ratios) of each source, and the isotopic composition of the two extreme sulphide ore samples from the Laisvall mixing line.

Our work provides direct evidence that petroleum is not only the main source of sulphate reductants for TSR, but can also transport significant amounts of metals that may end up as sulphides in base metal sediment-hosted ore deposits. The high proportion of lead contributed to the Laisvall deposit by petroleum and associated waters opens the possibility to evaluate in a new way the role of hydrocarbons in genetic models for epigenetic sediment-hosted base metal (e.g., Cu, Pb, Zn) ore deposits.

## Methods

Samples, weighing 1 to 4 kg each, were collected from drill cores at the core archive of Boliden Mineral AB, Sweden or from outcrops in a 5-km radius around the Laisvall mine: (i) shale hydrocarbon source rocks from the Alum Shale Formation (*n* = 5), (ii) mineralized sandstone samples (*n* = 47), (iii) basement granite samples (*n* = 6). Each drill core piece was cleaned in acetone and deionized water prior to processing. K-feldspar aliquots (*n* = 7) from the granite samples were prepared using a microdrill device.

The five shale hydrocarbon source rock samples and four mineralized sandstone samples were crushed, pulverized (>125 µm) and c. 200 g aliquots were taken for organic geochemistry studies. The extractable organic matter (EOM) was isolated from these aliquots by reflux in a mixture of dichloromethane-methanol and using petroleum geochemical procedures^9,35^ carried out at the Stable Isotope and Organic Geochemistry Laboratories at the University of Lausanne, Switzerland. EOM did not contain any non-organic material (e.g., clays, other silicates, carbonates, oxides, phosphates).

The EOM from the shale hydrocarbon source rocks (15-ASF-1 to 15-ASF-5) and mineralized sandstone samples (15-US-01 & -02, 15-LS-01 & -02) was processed in the following way. A new protocol was designed to extract the organo-metallic fractions of the EOM with the aim to obtain lead isotopic compositions. Following filtration, all dichloromethane solutions containing EOM were transferred to 5 mL glass vials in which solvent evaporation proceeded for two weeks. Depending on the amount of EOM present in the vials after solvent evaporation, between 1 and 1.5 mL of ultrapure 96% sulphuric acid (Pb content < 100 ppt) was added to each glass vial. This step produced (i) an ionic and aqueous leachate by oxidation of organo-metallic compounds in the EOM and (ii) a solid residue. Leachates were collected and converted to bromide form to perform standard Pb ion-exchange chromatography. In addition, four other mineralized sandstone samples and an aliquot of the EOM of alum shale sample 15-ASF-3, which was itself split in three subaliquots were utilized to determine the metal contents (i.e., U, Th, Pb, Zn) of their bitumen fractions by XRF. The metal contents (e.g., U, Th, Pb, Zn) of EOM in these aliquots were analysed at Weatherford Laboratories in Shenandoah, Texas, USA.

Whole rock samples of basement granite and corresponding K-feldspar aliquots were processed following the protocol of total extraction proposed by ref.^33^; the Pb isotopic compositions of HCl-HNO_3_ leachate and residue from HF-HNO_3_ bulk dissolution were obtained from these granite samples and K-feldspar aliquots.

Pure galena aliquots from mineralized sandstone samples (*n* = 42) were dissolved in 14 N HNO_3_ and dried down. Additional lead aliquots were obtained from sphalerite samples at the Laisvall deposit (*n* = 6) during Rb-Sr-Pb column chemistry^15^. This dataset was complemented with published Pb isotope data of galena and sphalerite aliquots from vein occurrences in the Palaeoproterozoic basement^31^.

All dried down Pb elutions (EOMs, HCl-HNO_3_ leachates, HF-HNO_3_ residues, galena aliquots) were re-dissolved using 5 mL 2% HNO_3_. Measurements of Pb isotopic composition were carried out on a Neptune Plus MC-ICPMS at the Section of Earth Sciences, University of Geneva, Switzerland, by adding Tl to the sample solution and using the ^203^Tl/^205^Tl ratio (0.418922) to correct for internal mass fractionation. ^204^Hg interference on ^204^Pb was corrected by monitoring ^202^Hg. The Pb isotope ratios were also corrected for external fractionation to the values of the SRM981 standard^36^. The long-term reproducibility of Pb isotope ratios is 0.0048% for ^206^Pb/^204^Pb, 0.0049% ^207^Pb/^204^Pb, and 0.0062% ^208^Pb/^204^Pb.

The slopes with positive and negative uncertainties (2σ) of the respective lines in a conventional ^207^Pb/^204^Pb vs. ^206^Pb/^204^Pb diagram for sulphides from Laisvall on the one hand, and, from the combined vein occurrences on the other hand were determined using a robust, nonparametric regression that makes no assumptions about the cause(s) of the observed scatter of the data from a straight line, and that requires no arbitrary decisions about what data should or should not be included in the regression^37^.

## Supplementary information

Tables SI1 to SI5

## Data Availability

The authors declare that the data supporting the findings of this study are available within the paper and its Supplementary Information files.
